# Differences in the Cyanobacterial Community Composition of Biocrusts From the Drylands of Central Mexico. Are There Endemic Species?

**DOI:** 10.3389/fmicb.2019.00937

**Published:** 2019-05-03

**Authors:** Itzel Becerra-Absalón, M. Ángeles Muñoz-Martín, Gustavo Montejano, Pilar Mateo

**Affiliations:** ^1^Departamento de Biología, Facultad de Ciencias, Universidad Autónoma de Madrid, Madrid, Spain; ^2^Departamento de Biología Comparada, Facultad de Ciencias, Universidad Nacional Autónoma de México, Mexico City, Mexico

**Keywords:** biological soil crusts, cyanobacteria, Illumina sequencing, drylands, endemic species

## Abstract

In drylands worldwide, biocrusts, topsoil microbial communities, are prevalent, contributing to the biostabilization of soils and allowing the subsequent establishment and growth of vascular plants. In early successional biocrusts, cyanobacteria are the first dominant colonizers of bare ground, largely determining their functioning. However, there are large gaps in our knowledge of the cyanobacterial diversity in biocrusts, particularly in understudied geographic regions, such as the tropical latitudes. We analyzed the diversity of the cyanobacteria inhabiting the biocrusts of semideserts from Central Mexico in two localities belonging to the same desert system (Chihuahuan Desert) that are separated by a cordillera that crosses the center of Mexico. Morphological identification of the cyanobacteria was carried out after cultivation in parallel with the direct observation of the environmental samples and was supported by genetic characterization through analysis of the 16S rRNA gene of the isolated strains and by next-generation sequencing of the soil samples. Taxonomic assignment revealed a clear dominance of heterocystous cyanobacteria at one of the studied locations (Actopan, Hidalgo state). Although heterocystous forms were abundant at the other location (Atexcac, Puebla state), almost a third of the cyanobacterial phylotypes were represented by unicellular/colonial cyanobacteria, mostly *Chroococcidiopsis* spp. Only 28.4% of the phylotypes were found to be common to both soils. Most of the other taxa, however, were biocrust-type specific, and approximately 35% of the phylotypes were found to be unique to the soil they were collected in. In addition, differences in the abundances of the shared cyanobacteria between the locations were also found. These differences in the cyanobacterial distribution were supported by the distinct responses of the isolated strains representative of the sites to extreme heat and desiccation in bioassays. Some cyanobacteria with high abundance or only present at the hottest Actopan site, such as *Scytonema hyalinum*, *Scytonema crispum, Nostoc commune*, *Nostoc* sp., and *Calothrix parietina*, survived extreme heat and desiccation. However, *Tolypothrix distorta* and *Chroococcidiopsis* spp. were clearly sensitive to these extreme conditions in relation to their lower abundances at Actopan as opposed to Atexcac. Since novel biocrust-associated phylotypes were also found, the emergence of endemic cyanobacterial taxa is discussed.

## Introduction

Biological soil crusts (biocrusts) are assemblages of different organisms (principally cyanobacteria, heterotrophic bacteria, algae, fungi, lichens, and bryophytes) that develop in dryland ecosystems compromising the topsoil layer, in which soil particles are aggregated through the presence and activity of these biota, and the resulting living crust covers the surface of the ground in the interspaces between plants (Garcia-Pichel, 2002; Belnap and Lange, 2003). Cyanobacteria have been proposed to act as pioneers in the stabilization process of these soils (Garcia-Pichel and Wojciechowski, 2009), with the production of polysaccharide sheaths by some of them, that aid in the formation of often centimeter-long filament bundles, contributing to the creation of a layer of fines and further enabling secondary colonization by heterocystous cyanobacteria, with eventual colonization by lichens or mosses (Garcia-Pichel et al., 2016). Soil characteristics, mainly texture, greatly influence biocrust formation and structure since a fine texture allows motility of colonizing filaments, further increasing soil aggregation (Rozenstein et al., 2014; Chamizo et al., 2018). The physiological features of different cyanobacteria will determine the colonizing succession within a cyanobacterial biocrust community, since those populations able to cope to extreme conditions, such as desiccation, temperature extremes, and high radiation, will survive in these unfavorable environments, such as the drylands. Many of these traits are related to adaptive mechanisms, for instance, the synthesis of sunscreen pigments or dormant cells (akinetes) that allow to cope with high radiation and desiccation respectively (Garcia-Pichel and Castenholz, 1991; Hu et al., 2012). Evidence has shown that cyanobacteria from biocrusts play important roles in key ecosystem processes, such as their contributions to soil fertility through nitrogen fixation and carbon sequestration, their ability to stabilize soils, their response to and recovery from fire and surface disturbance, and their effects on vascular plant establishment and growth (Lange and Belnap, 2016). Nitrogen-fixing cyanobacteria, free or growing in symbiosis in lichens, are the dominant N fixers in biocrusts (Barger et al., 2016), which exert a strong influence on the enrichment of the N pool in soils from low-nutrient environments.

Biocrusts are found on all continents, and studies of their distribution have expanded in North America, Asia, Africa, the Middle East, and Europe (Bowker et al., 2016 and references therein). Knowledge about cyanobacteria is rapidly increasing with the use of culture-independent sequencing studies. For instance, a continental-scale compositional survey of the cyanobacterial diversity in biocrusts of geographically distinct areas in the arid western United States revealed patterns of its abundance and distribution (Garcia-Pichel et al., 2013). Other studies showed differences in the cyanobacterial populations depending on the geographical region. In different arid and semiarid regions of warm climates, the filamentous non-heterocystous genus *Microcoleus* is the major component of the biocrusts (Büdel et al., 2016), while in Western Europe and Artic biocrust other filamentous non-heterocystous cyanobacteria belonging to the family of Leptolyngbyaceae were found to be dominant (Pushkareva et al., 2015; Williams et al., 2016). Less variation has been found regarding heterocystous cyanobacteria, whereby the genera *Scytonema*, *Nostoc*, and *Tolypothrix* have been found as dominant worldwide (Büdel et al., 2016). However, few investigations have been undertaken in temperate regions, and very little information is available from tropical latitudes (Belnap and Lange, 2003; Rivera-Aguilar et al., 2006; Büdel et al., 2009; Castillo-Monroy et al., 2011). In the central region of Mexico, there are arid and semiarid zones comprising important ecosystems, covering approximately 60% of the territory, in which biocrusts have a wide distribution that can constitute up to 70% of the drylands (Montaño and Monroy, 2000; Rivera-Aguilar et al., 2004). Therefore, in this work, two semidesert localities from Central Mexico were selected, one in Hidalgo state and another in Puebla state. These localities constitute the southern end of the oldest and most stable desert in Mexico, aged at approximately 50 million years old, the Chihuahua Desert. We aimed to study the differences in cyanobacterial diversity in biocrusts from these two locations, with distinct microclimatic conditions, through a polyphasic strategy in which microscopic analysis and molecular sequencing were combined with bioassays in order to identify causal factors influencing cyanobacterial species composition. This combined approach allowed us to determine the cyanobacterial diversity of biocrusts from one of many countries that is known for having extensive areas where these types of ecosystems are dominant, contributing to our understanding of the identity and distribution of these keystone microorganisms.

## Materials and Methods

### Sites for Collection of Biocrust Samples

**FIGURE 1:**
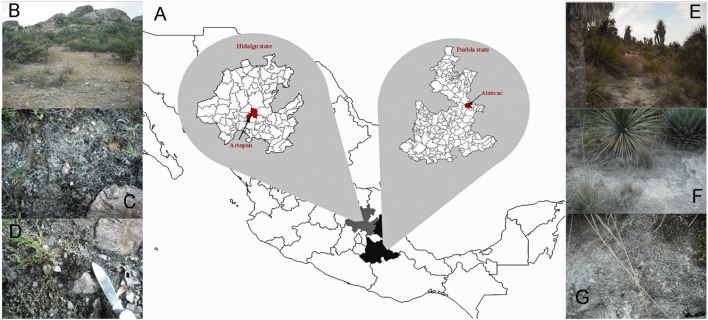
**(A)** Location of sampling sites, **(B)** general view of Actopan locality in Hidalgo state, **(C,D)** biocrusts from Actopan, **(E)** general view of Atexcac locality in Guadalupe Victoria, Puebla state, **(F,G)** biocrusts from Atexcac.

**FIGURE 2:**
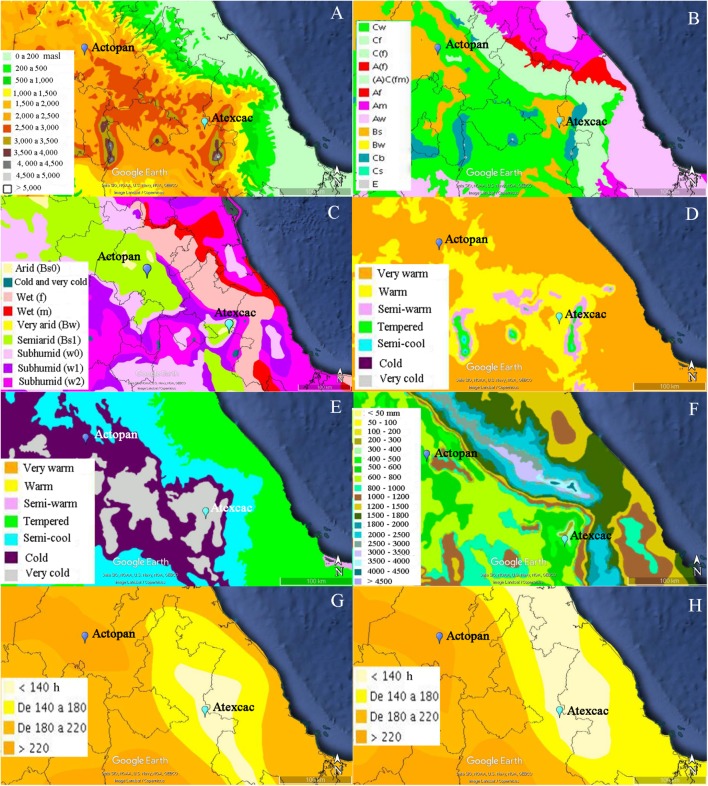
Hypsometry and climatic characteristics of the two sampling sites: **(A)** hypsometry, **(B)** climate, **(C)** humidity, **(D)** maximum mean annual temperature, **(E)** minimum mean annual temperature, **(F)** mean annual precipitation, **(G)** hours of insolation during the month of maximum insolation (May), **(H)** hours of insolation during the month of minimum insolation (January). The climatic and geological data were obtained from the Geoportal of the National Commission for the Knowledge and Use of Biodiversity (CONABIO) in KML format and then imported into Google Earth (based on Image Landsat/Copernicus-Data SIO, NOAA, U.S. Navy, NGA, GEBCO) where the sites were located by means of the coordinates obtained with a GPS. masl, meters above sea level; Cw, temperate subhumid with dry winter; Cf, temperate humid; C(f), temperate without dry season; A(f), hot humid; (A)C(fm), semi-hot humid; Af, hot humid; Am, hot with summer precipitations; Aw, wet; Bs, semi-arid steppe; Bw, arid desert; Cb, temperate warm summer; Cs, temperate, dry summer; E, polar.

Biocrusts were collected from two semidesert localities from Central Mexico belonging to the extreme south of the Chihuahuan Desert (Rzedowski, 2006) (Figure 1). The first site was near the Actopan, Hidalgo state (20°16′02.9″N; 98°54′57.5″W) within the Mezquital Valley, and the second location was around the crater lake of San Luis Atexcac, Puebla state (19°20′13″N; 97°21′19″W) (Figure 2). Basaltic volcanic flows and mountains (Figure 2A) separate the two sites which are located in tropical latitudes. However, due to the altitude at which they are located, more than 2,000 m.a.s.l., they are considered temperate zones. The rainfall regime is different from that of the tropical latitude due to the development of orographic rain shadows (Rzedowski, 1978) so the climate is semiarid (Figures 2B,C). There are two main seasons, a warmer one, from May to October and a cooler one, during the rest of the year. In the warmest month (May), the mean monthly temperature in Actopan was 19.1°C (average data from 1981 to 2010), and the minimum and maximum average temperatures ranged from 9.5 to 28.7°C; while in Atexcac, this was 17.0°C, and ranged from 7.7 to 26.3°C. In the coldest months (January and December) the mean monthly temperature in Actopan was 12.4°C and ranged from 2.5 to 22.2°C, while in Atexcac this was 11.2°C and ranged from 1.3 to 21.1°C. The rainfall mainly occurs in the warmer season, normally in June and September. The Actopan location presented a mean annual temperature (MAT) of 16.4°C, the minimum and maximum mean annual temperatures were 7.8°C and 25.1°C, respectively, and the minimum and maximum temperatures recorded on the coldest and warmest days were -7°C and 47°C, respectively. The mean annual precipitation (MAP) was 436 mm, and the precipitation ranged from 400 to 900 mm. The soil type was a Phaeozem (Mollisol), fine-textured, with both alluvial and andesita deposits, and the vegetation was xerophytic crassicaule shrubs (Mesquite shrubs and trees), dominated by *Prosopsis* spp. with crassicaule (fleshy stemmed) plants. The Atexcac location had an MAT of 13.9°C, the minimum and maximum annual temperatures were 5.5°C and 22.4°C, respectively, and the minimum and maximum temperatures recorded on the coldest and warmest days were -12°C and 36°C, respectively. The MAP was 372 mm, with a precipitation range of 400–900 mm. The soil type was Phaeozem calcareous, fine-textured, and the vegetation was xerophytic shrubs with Izotal, dominated by *Yucca* and other Agavoideae (Alcocer et al., 2004). Another important climatic difference between the sites was the insolation; in Actopan, the location receives greater insolation all year round than does Atexcac (Figures 2G,H). The environmental data were obtained from government databases at Servicio Metereológico Nacional (SMN^1^, accessed March 10, 2017), Instituto Nacional de Estadistica y Geografía (INEGI^2^, accessed March 10, 2017), and Geoportal de la Comisión Nacional para el Conocimiento y Uso de la Biodiversidad (Conabio^3^, accessed March 10, 2017) and had been recorded over many years of monitoring (1951–2019). Daily climatic data of the SMN were obtained through the CICESE web platform^4^.

### Biocrust Sampling

Biocrust samples were collected in October (Actopan), and December (Atexcac) 2014 following the procedure previously described (Garcia-Pichel et al., 2013; Muñoz-Martín et al., 2019): Nine biocrust samples were taken in each site, within an area of 25–50 m^2^ that was inspected to choose places that had developed biocrusts. A preliminary determination of the major cyanobacterial morphotypes and their relative abundance was carried out by direct microscopy of wetted samples. Then, representative subsamples, according to microscopic inspection, and with equal size, were selected, mixed together and homogenized with a mortar and pestle, to form a composite sample. These multiple samples, integrated field patchiness of the communities (Pushkareva et al., 2015; Muñoz-Martín et al., 2019). The samples were collected using 60 mm Petri dishes, of which the plate bottom was inserted into the surface to excise a circular portion of the biocrust, collecting approximately 1 cm deep soil without disturbing the upper layer. After collection, the samples were stored in the dark at room temperature until use.

### Strains Isolation and Culture Conditions

The dry biocrusts were reactivated by the addition of distilled water before their use for the isolation of strains. We isolated the strains by two methods: one by micromanipulation of the samples under the dissecting microscope with watchmaker’s forceps, in which we manually isolated bundles of filaments as previously described (Garcia-Pichel et al., 2013) as well as filaments of heterocystous cyanobacteria. Seven strains were isolated by this procedure (see Table 1). The isolated bundles or filaments were then inoculated in multiwell plates with liquid BG11 (for non-heterocystous cyanobacteria) or BG11_0_ medium (for heterocystous cyanobacteria) (Rippka et al., 1979). Cycloheximide (0.1 mg mL^-1^) was also added to avoid fungal contamination. In a second, parallel method, the topsoil samples were ground with a mortar, and 0.1 g was mixed with 1.5 mL of cyanobacterial culture media and inoculated into Petri dishes (1.5% agar, with cycloheximide 0.1 mg mL^-1^) with BG11 or BG11_0_ media. The samples were incubated in a growth chamber at 28°C, 20–50 μmol photon m^-2^ s^-1^ and allowed to grow for approximately 4 weeks. Each strain was isolated from the colonies by selecting single trichomes or groups of cells for unicellular cyanobacteria using pulled capillary pipettes or forceps under a dissecting microscope (Leica, Leica Microsystems, Wetzler, Germany). The isolated strains were transferred to multiwell plates with liquid BG11 or BG11_0_ and maintained under 28°C, 20–50 μmol photon m^-2^ s^-1^.

**Table 1:** Cyanobacterial strains isolated in this study.

	Strain number/culture	Sampling
Taxon	collection no.	site	Figure
*Nostoc commune*	ACT709/UAM422	Actopan	Figure 3A
*Nostoc* sp.1	ACT703/UAM857	Actopan	Figure 3B
*Nostoc* sp.2	ACT732/UAM446	Actopan	Figure 3C
*Tolypothrix distorta*	ACT712/UAM443	Actopan	Figure 3E
	ATE705/UAM438	Atexcac	Figure 3D
	ATE717/UAM454	Atexcac	
*Scytonema crispum*	ACT685/UAM437^∗^	Actopan	Figure 3F
*Calothrix parietina*	ACT696/UAM439	Actopan	Figure 3G
	ACT713/UAM858	Actopan	
*Scytonema hyalinum*	ACT694/UAM441	Actopan	
	ACT695/UAM447	Actopan	
	ATE698/UAM456	Atexcac	
	ACT699/UAM440^∗^	Actopan	Figure 3H
	ACT700/UAM436	Actopan	
	ATE704/UAM455^∗^	Atexcac	Figure 3I
	ACT706/UAM859^∗^	Actopan	
	ACT711/UAM448^∗^	Actopan	
	ATE714/UAM860	Atexcac	
*Chroococcidiopsis* sp.1	ATE715/UAM434	Atexcac	Figure 3J
*Chroococcidiopsis* sp.2	ATE707/UAM433	Atexcac	Figure 3K
*Kamptonema* sp.	ACT692/UAM457	Actopan	Figure 3M
*Microcoleus vaginatus*	ACT688/UAM445^∗^	Actopan	Figure 3N
*Porphyrosiphon notarisii*	ACT693/UAM430^∗^	Actopan	Figure 3O
*Oculatella atacamensis*	ATE710/UAM427	Atexcac	Figure 3P
*Oculatella* sp.	ACT687/UAM426	Actopan	Figure 3V
*Leptolyngbya* sp.	ACT691/UAM424	Actopan	Figure 3T
*Leptolyngbya* sp.	ACT689/UAM432	Actopan	Figure 3U
*Leptolyngbya frigida*	ACT684/UAM425	Actopan	Figure 3Q
*Schizothrix* sp.	ACT690/UAM431	Actopan	Figures 3R,S
*Chroakolemma opaca*	ACT686/UAM423	Actopan	
	ACT701/UAM861	Actopan	Figure 3W
	ACT702/UAM862	Actopan	
	ACT708/UAM863	Actopan	
*Chroakolemma pellucida*	ATE716/UAM429	Atexcac	
	ATE718/UAM428	Atexcac	
	ATE719/UAM864	Atexcac	Figure 3X
*Synechococcus* sp.	ATE729/UAM435	Atexcac	Figure 3L


*^∗^Strains isolated by micromanipulation (see “Materials and Methods” section).*

### Morphological Characterization

The isolated cultured strains and cyanobacterial populations in the environmental samples were analyzed for general morphological and morphometric characteristics as previously described (Becerra-Absalon et al., 2018). The characteristics of our cyanobacteria were compared with the information provided in the taxonomic keys of Komárek and Anagnostidis (1999, 2005) and Komárek (2013).

### Isolation of Genomic DNA and Amplification of the 16S rRNA Gene of the Isolated Strains and an Environmental Sample

Total genomic DNA extraction of the isolated cultures and an environmental sample of a macroscopic colony of *Nostoc* was accomplished using the Ultraclean^®^ Microbial DNA Isolation Kit (Mo Bio Laboratories, Inc., Carlsbad, CA, United States) with a modification previously described (Loza et al., 2013) to break the exopolysaccharides surrounding many of the cyanobacterial cells. This involved a three-cycle step that consisted of freezing 0.3 mL aliquots of cyanobacterial suspensions of each culture in liquid nitrogen, breaking them down with an adapted drill and melting them in a 60°C water bath.

The 16S rRNA gene was amplified by PCR using primer 27 as the forward primer (Wilmotte et al., 1993) and primer B23SR as the reverse (Lepère et al., 2000) under conditions previously described by Mateo et al. (2011) and following the PCR conditions of Gkelis et al. (2005). This reaction produced amplification fragments of approximately 2,000 bp that span the 16S rRNA gene and the intergenic region between the 16S and 23S rRNA genes. An agarose gel (1%) with a 1 kb gene ruler (MBL Biotools, Spain) and the fluorescent DNA stain Gel Red^TM^ were employed to check if the amplification fragments had the correct size. The DNA was purified using Wizard SV Gel and PCR Clean-Up (Promega, Madison, WI, United States) and cloned into pGEM^®^-T Easy Vector Systems (Promega, Madison, WI, United States). The DNA from the colonies of recombinant clones carrying the correct-sized inserts was extracted using the Wizard Miniprep Kit (Promega). The sequencing of both strands was carried out at the Genomics Core Unit of the Spanish National Cancer Research Center, Spain. Several clones from various strains were sequenced, obtaining a total of 46 nucleotide sequences which were deposited in the GenBank Database under the accession numbers: MK247967-MK248010 (isolated strains) and MK239479-MK239480 (environmental sample).

### Phylogenetic Analyses of Sequence Data

The 16S rRNA gene sequences of approximately 1,500 bp were aligned and manually corrected using PhyDE-1 v0.9971 (Müller et al., 2010). A BLAST search (Altschul et al., 1990) was performed, and assignations with an identity value higher than 97.5% and other representative soil cyanobacteria sequences were downloaded from the NCBI database^5^. Multiple alignments of all these sequences, using *Gloeobacter violaceus* or *Chroococcidiopsis* spp. sequences as outgroups, were accomplished using the function ClustalW multiple alignment, and the alignment was later visually checked and corrected using PhyDE-1 v0.9971 (Müller et al., 2010). The phylogenetic trees were computed with MEGA version 7.0.21 (Kumar et al., 2016) using neighbor-joining (NJ), maximum parsimony (MP) and maximum likelihood (ML) algorithms. For NJ, the evolutionary distances were calculated by applying the Kimura 2-parameter, gamma distributed and invariant sites with a pairwise deletion of gaps and missing data. The distances for the ML tree were estimated by the Kimura 2-parameter, gamma distributed and invariant sites, assuming a gamma distribution with four categories with the nearest-neighbor-interchange. The MP tree was built with the subtree-pruning-regrafting search method with 10 initial trees and 3 search levels. The gaps and missing data were treated with the complete deletion option. The standard error in all analyses was estimated with the bootstrap phylogeny test (Felsenstein, 1985) using 1,000 replications. The percentage of similarity between sequences was determined as (1-p-distance)^∗^100.

### Analyses of Cyanobacterial Community Composition by Amplicon Metagenomics

Amplicon metagenomics targeted to the 16S rRNA gene and Illumina MiSeq sequencing data were used to assess the diversity and community composition of the bacteria. One gram from each composite soil sample was aliquoted into four parts (0.25 g) for DNA extraction using a PowerSoil DNA extraction Kit (Mo Bio, Carlsbad, CA, United States), according to the manufacturer’s instructions. Because many cells were difficult to lyse, an additional step was added at the beginning of the protocol as described in Muñoz-Martín et al. (2019): the soil was incubated with the homogenization solution and exposed to three freeze-thaw cycles, alternating immersion in liquid nitrogen, heating to 60°C, and homogenizing with a pellet pestle in an Eppendorf tube using a hand-operated homogenizer (Bosch, CSB-850-2RET). The DNA was eluted in 100 μL of buffer, and the four independent DNA extractions per sample were mixed for PCR amplifications. The V4 variable region from the 16S rRNA gene was amplified by PCR using the universal bacterial primers F515 and R806 as previously described by Caporaso et al. (2011) at the Microbiome Analysis Laboratory, Swette Center for Environmental Biotechnology, Biodesign Institute of the Arizona State University (United States). The amplicons were processed using a MiSeq sequencer (Illumina) with a read length of 2 × 150 bp. At least 100,000 sequences were obtained for each amplicon. Quality control checks were performed on the raw sequence data using FastQC v 0.11.3. The sequence data were processed using QIIME v 1.9.0 (Caporaso et al., 2010) and the workflow described by Pylro et al. (2014), available on http://www.brmicrobiome.org/, based on UPARSE pipeline (Edgar, 2013) implemented by the software USEARCH v. 8.1, as described in detail by Muñoz-Martín et al. (2019). A similarity cut-off value of 97% was used to cluster the operational taxonomic units (OTUs). The OTU representative sequences were taxonomically assigned against the Greengenes database (13-08) (McDonald et al., 2011) first, using the RDP classifier method with a confidence value of 0.8 (Navas-Molina et al., 2013) to define the bacterial community composition at the phylum level. For more accurate taxonomic assignation of the cyanobacterial community, an approach similar to that described in Muñoz-Martín et al. (2019) was followed. First, the representative OTU sequences were matched against the 16S rDNA sequence database of the cultures obtained in this study, sequences from other cultures obtained by us from other biocrusts and other OTUs obtained in the abovementioned paper using the “uparse ref command” in USEARCH with the default parameters (Edgar, 2010). Then, all this information was compared with the taxonomic assignments made against the Greengenes database (McDonald et al., 2011) as mentioned above, and also against the SilvaMod database (Yilmaz et al., 2014) using the lowest common ancestor (LCA) algorithm implemented in CREST (Lanzen et al., 2012). In addition, the OTU representative sequences were Blasted against NCBI database. These steps allowed us to taxonomically assign almost all of the OTUs with a relative abundance of more than 0.1% in any of the locations. Alpha diversity indices (Chao1 estimator, Good’s coverage and observed OTUs) were calculated using QIIME. Good’s coverage estimates ranged from 99.73 to 99.80%, indicating that the large majority of the cyanobacterial diversity was captured. The OTU sequences have been deposited in the GenBank under accession numbers MK247058-MK247141. Raw sequencing data have been deposited in the NCBI Sequencing Read Archive under accession number PRJNA507723.

### Cyanobacterial Survival Bioassays

To determine survival at high temperature and desiccation, a set of experiments was carried out with 23 of the isolated strains (see Table 4 in the results). The survival was tested by two ways: the resistance (the ability of the cyanobacteria to withstand the extreme conditions), and the resilience (the ability of the cyanobacteria to recover following the extreme conditions). Therefore, first, bioassays were carried out at 40°C in liquid BG11 or BG11_0_ (for non-heterocystous and heterocystous cyanobacteria, respectively) (Rippka et al., 1979) with equal amounts of inoculum from each of the strain, in duplicate; strains were distributed in wells of sterile polystyrene 6-well microtiter plates (IWAKI Microplate). They were incubated for 25 days in a 16:8 h light:dark period with an irradiance of 30 μmol photon m^-2^s^-1^. After this period of time, the cultures were left in these conditions until total desiccation and maintained thereafter for a year. To test the resilience following these conditions, culture medium was added to the desiccated strains and then the samples were maintained at room temperature (22–24°C). The retention or total loss of pigmentation was tested for these bioassays, using the retention of chlorophyll a as a proxy of the survival, as previously described (Garcia-Pichel et al., 2013; Zhou et al., 2016).

## Results

### Morphological and Molecular (16S rRNA Gene) Analysis of Cultures: Polyphasic Identification of Isolated Strains

**FIGURE 3:**
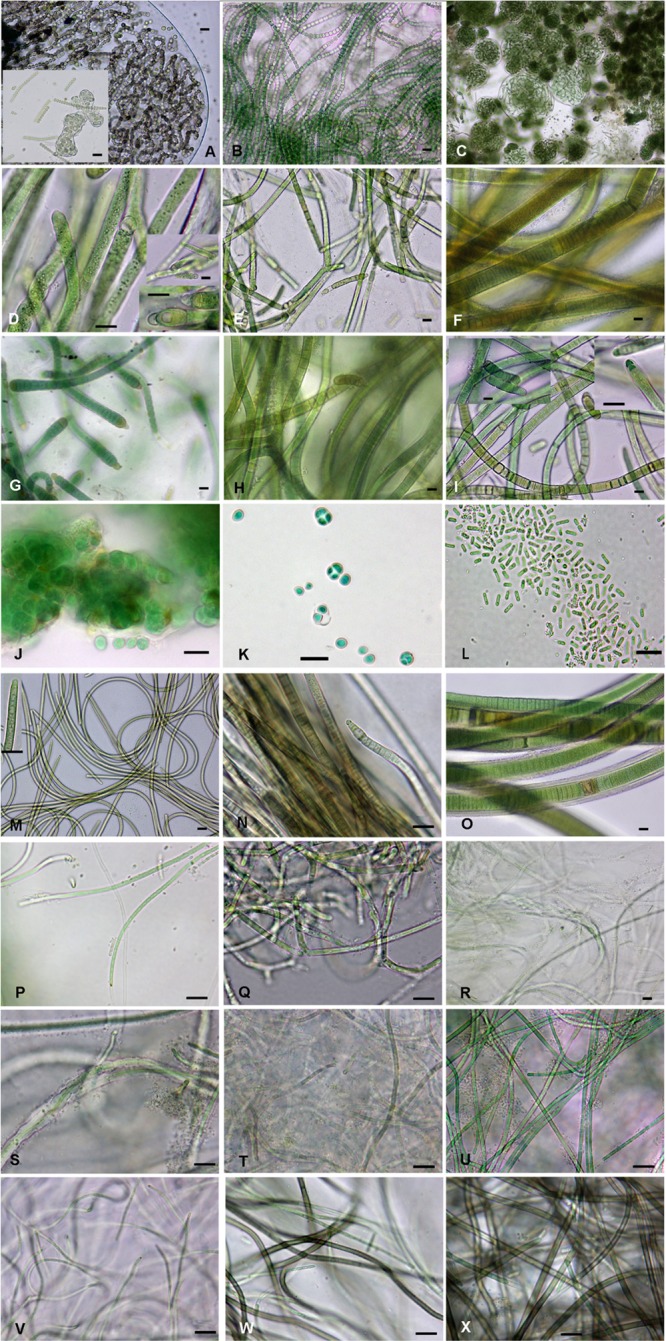
Cyanobacterial isolated strains: **(A)** *Nostoc commune* ACT709, **(B)** *Nostoc* sp. 1 ACT703, **(C)** *Nostoc* sp. 2 ACT732, **(D)** *Tolypothrix distorta* ATE705, **(E)** *Tolypothrix distorta* ACT712, **(F)** *Scytonema crispum* ACT685, **(G)** *Calothrix parietina* ACT696, **(H)** *Scytonema hyalinum* ACT699, **(I)** *Scytonema hyalinum* ATE704, **(J)** *Chroococcidiopsis* sp.1 ATE715, **(K)** *Chroococcidiopsis* sp.2 ATE707, **(L)** *Synechococcus* sp. ATE729, **(M)** *Kamptonema* sp. ACT692, **(N)** *Microcoleus vaginatus* ACT688, **(O)** *Porphyrosiphon notarisii* ACT693, **(P)** *Oculatella atacamensis* ATE710, **(Q)** *Leptolyngbya frigida* ACT684, **(R,S)** *Schizothrix* sp. ACT690, **(T)** *Leptolyngbya* sp.1 ACT691, **(U)** *Leptolyngbya* sp.2 ACT689, **(V)** *Oculatella* sp. ACT687, **(W)** *Chroakolemma opaca* ACT701, **(X)** *Chroakolemma pellucida* ATE 719. Scale bar: 10 μm.

**FIGURE 4:**
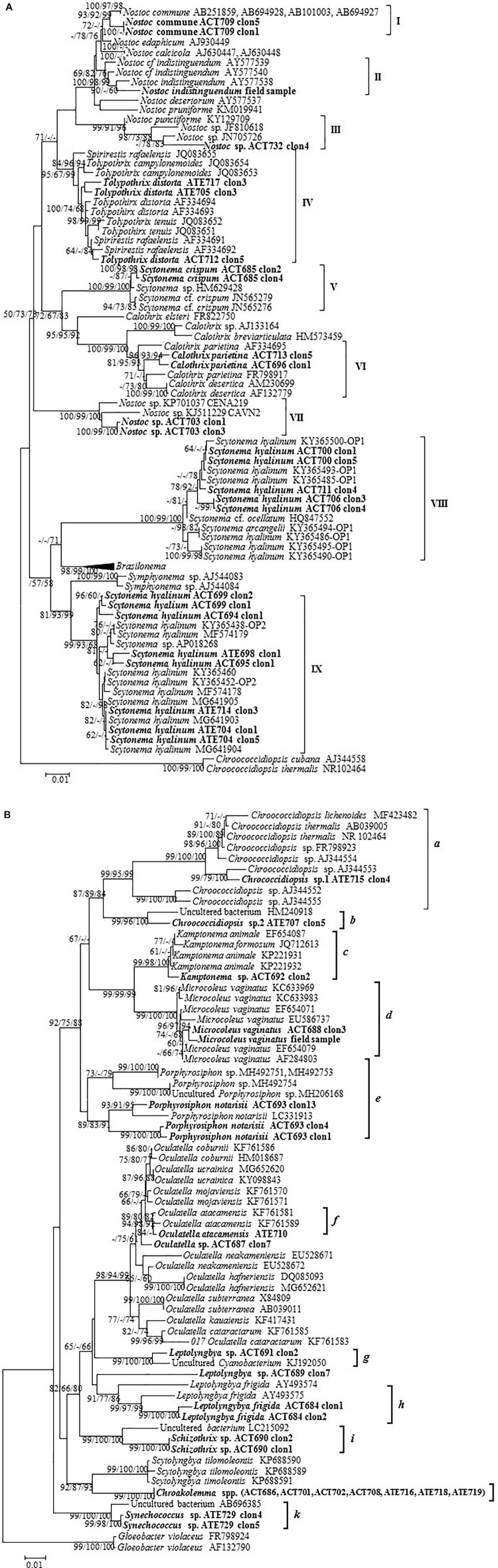
Phylogenetic trees obtained by the neighbor-joining method representing **(A)** heterocystous cyanobacteria and **(B)** unicellular and filamentous non-heterocystous cyanobacteria, based on the analysis of the 16S rRNA gene, showing the position of the sequences obtained from the present study (in bold). Numbers near nodes indicate bootstrap values greater than or equal to 60% for NJ, ML, and MP.

A total of 37 strains were isolated from the biocrust samples and cultured (Table 1); these strains were phenotypically and phylogenetically characterized (Figure 3, 4). In addition, an environmental sample of a macroscopic colony of *Nostoc* found at the Actopan sampling site and was also characterized to com- pare with the *Nostoc* isolated strains (see below). The combined morphological and genetic evaluation allowed us taxonomic identification of 21 cyanobacterial species (Table 1), in which, strains corresponding to the same species showed similar mor- phological characteristics (data not shown). Eighteen of the strains belonged to the heterocystous cyanobacteria (Figures 3A–I, 4A), and 19 were non-heterocystous, of which most were filamentous (16 strains), while only three strains belonged to unicellular/colonial cyanobacteria (Figures 3J–X, 4B, 4B). Nine clusters were identified in the phylogenetic tree of the heterocystous cyanobacteria (Figure 4A) and corresponded to the morphotypes found (Figures 3A–I). The *Nostoc* isolates fell into three different clusters (cluster I, III, and VII) according to their distinct morphological characteristics (Figures 3A–C). The 16S rRNA gene sequence from the *N. commune* isolate was placed in a cluster gathering of known representatives of *N. commune* from soils (cluster I). However, the sequences corresponding to the other isolated strains of the genus *Nostoc* (*Nostoc* sp. ACT732 and *Nostoc* sp. ACT703) were located in separate clusters, each of which included representatives of this genus, but with no clear specific identification (clusters III and VII, respectively). All the *Nostoc* phylotypes from the isolated strains were separated from those in cluster II, which included the sequence of the environmental sample fitting the morphological characteristics of *Nostoc indistinguendum* as well as other representatives of this taxon (cluster II). The well-supported group (cluster IV) corresponding to *Tolypothrix/Spirirestis* included our three isolates of *Tolypothrix distorta*, which showed typical characteristics of this genus, such as single falsely branched filaments originating below intercalary heterocysts (Figures 3D,E). Similarly, the morphological characteristics of the strain fitting the descriptions of *Scytonema crispum* (Figure 3F) were supported by its inclusion in a cluster together with other sequences of this taxon from the database (cluster V). Two isolates corresponding to *Calothrix* grouped with other *Calothrix* sequences from the databases corresponding to *C. parietina* (cluster VI); in addition, the morphological characteristics of these two isolates fit with those of this taxon (Figure 3G). The *Scytonema hyalinum* isolates (Figures 3H,I) also showed typical morphology of the genus, grouping with other sequences of this species in different clusters (clusters VIII and IX) corresponding to two divergent 16S rRNA operons from single strains, as previously found (Yeager et al., 2007; Johansen et al., 2017; Muñoz-Martín et al., 2019).

Regarding non-heterocystous cyanobacteria, unicellular/ colonial cyanobacteria were distributed in the phylogenetic tree among filamentous non-heterocystous cyanobacteria (Figure 4B) as previously found (Komarek et al., 2014). Clusters *a* and *b* included representatives of *Chroococcidiopsis* spp. (Figures 3J,K) together with sequences from this genus from databases (Figure 4B). The other coccoid cyanobacteria, which fitted into the genus *Synechococcus* (Figure 3L), were distantly placed from the *Chroococcidiopsis* spp. in the phylogenetic tree, with no matches found in the database (cluster *k*). Cluster *c* grouped our isolate identified as *Kamptonema* sp. (Figure 3M), a new genus derived from the polyphyletic genus *Phormidium* (Strunecky et al., 2014), with other sequences of this genus (Figure 4B). Typical filamentous cyanobacteria, usually included in taxonomic descriptions from biocrusts, were *Microcoleus vaginatus* (Figure 3N, cluster *d*), *Porphyrosiphon notarisii* (Figure 3O, cluster *e*), *Oculatella atacamensis* (Figure 3P, cluster *f*), and *Leptolyngbya frigida* (Figure 3Q, cluster *h*). In addition, novel phylotypes and/or morphotypes were found in the studied biocrusts: the 16S rRNA gene sequence from a *Schizothrix* sp. isolate mapped alone in the tree (Figures 3R,S, cluster *i*) since no similar sequences were found in the database (the most similar sequence belonged to an uncultured bacterium with only 96% similarity). The genera *Leptolyngbya* and *Oculatella* were also represented by other sequences with no close relatives (Figures 3T–V, 4B). Finally, seven of the isolated strains were recently characterized as belonging to a new genus: *Chroakolemma* (Becerra-Absalon et al., 2018) (Figures 3W,X, cluster *j*).

### Microscopic Observation of Biocrust Field Samples

**FIGURE 5:**
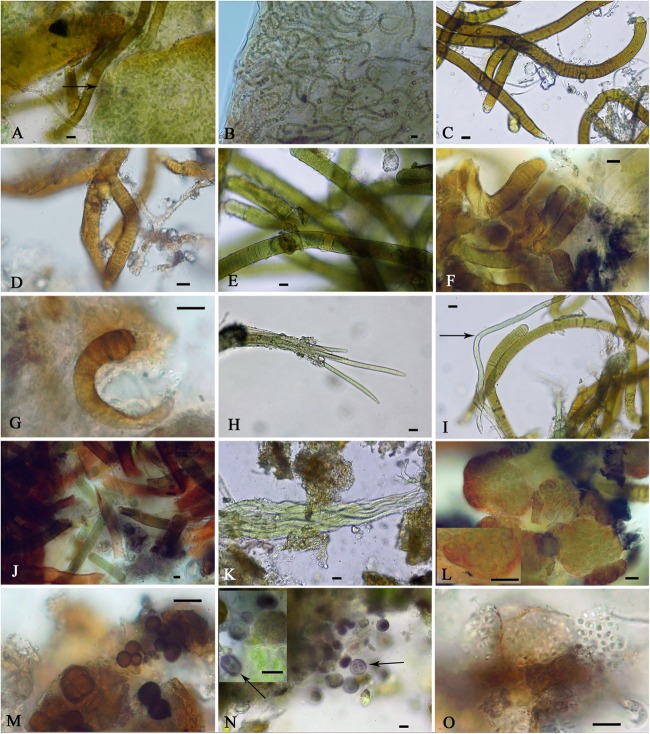
Cyanobacteria observed in field samples: **(A)** *Nostoc commune* (black arrow), **(B)** *Nostoc indistinguendum*, **(C)** *Scytonema hyalinum*, **(D)** *Tolypothrix distorta*, **(E)** *Scytonema crispum*, **(F)** *Dapisostemon apicaliramis*, **(G)** *Calothrix parietina*, **(H)** *Microcoleus vaginatus*, **(I)** *Chroakolemma opaca* (black arrow), **(J)** *Porphyrosiphon notarisii*, **(K)** *Symplocastrum flechtnerae*, **(L)** *Aphanocapsa* sp., **(M)** *Chroococcidiopsis* sp.1, **(N)** *Chroococcidiopsis* sp.2 (black arrow), **(O)** *Chlorogloea* sp. Scale bar: 10 μm.

The identification of the isolated strains allowed us to better analyze the cyanobacteria observed in the soil samples; however, not all of the isolated strains were observed in these samples which may be due to their presence in relatively small amounts, and some of the cyanobacteria found in the samples could not be isolated. Figure 5 shows representative micrographs of the cyanobacteria from the studied biocrusts, and Table 2 shows the locations of the taxa in the samples. Most of the cyanobacteria found presented envelopes and pigmentation that were characteristic of cyanobacteria found in these extreme environments (Figure 5). Typical heterocystous cyanobacteria from biocrusts, such as *N. commune*, *N. indistinguendum*, *S. hyalinum*, and *T. distorta* (Figures 5A–D, respectively), were observed at both sampling sites (Table 2). However, other heterocystous forms, such as *S. crispum, Dapisostemon apicaliramis* and *Calothrix parietina* (Figures 5E,F,G, respectively), were observed only at the Actopan sampling site (Table 2). Regarding non-heterocystous cyanobacteria, only *M. vaginatus* (Figure 5H) and *Chroakolemma* sp. (Figure 5I) were observed at both sampling sites, while *Porphyrosiphon notarisii* (Figure 5J), *Symplocastrum flechtnerae* (Figure 5K) and *Aphanocapsa* sp. (Figure 5L) were observed in the Actopan samples. *Chroococcidiopsis* representatives (Figures 5M,N) and *Chlorogloea* sp. (Figure 5O) were only found at Atexcac (Table 2).

**Table 2:** Cyanobacteria observed in field samples.

Taxon	Actopan	Atexcac	Figure
*Nostoc commune*	X	X	Figure 5A
*Nostoc indistinguendum*	X	X	Figure 5B
*Scytonema hyalinum*	X	X	Figure 5C
*Tolypothrix distorta*	X	X	Figure 5D
*Scytonema crispum*	X		Figure 5E
*Dapisostemonum apicaliramis*	X		Figure 5F
*Calothrix parietina*	X		Figure 5G
*Microcoleus vaginatus*	X	X	Figure 5H
*Chroakolemma* sp.	X	X	Figure 5I
*Porphyrosiphon notarisii*	X		Figure 5J
*Symplocastrum flechtnerae*	X		Figure 5K
*Aphanocapsa* sp.	X		Figure 5L
*Chroococcidiopsis* sp.		X	Figure 5M
*Chroococcidiopsis* sp.		X	Figure 5N
*Chlorogloea* sp.		X	Figure 5O


### Molecular Analysis of Microbial Community Composition

**FIGURE 6:**
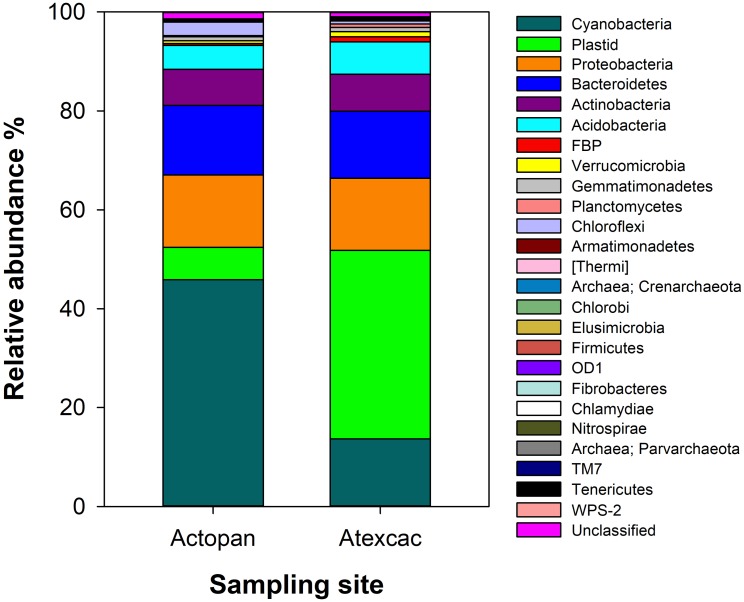
Taxonomic microbial community composition from the studied biocrusts.

The relative abundance of the dominant phyla of bacteria and plastids from the rRNA gene sequences of the studied biocrusts are displayed in Figure 6. The microbial phototrophs (cyanobacteria and algae) presented similar overall abundances in both samples (52%). However, this corresponded to a clear dominance of the cyanobacteria at Actopan (45.5%), while algae was the dominant phototroph community at Atexcac (38%). The rest of the biocrust bacterial rRNA gene sequences were also similar at both sampling sites; Proteobacteria and Bacteroidetes were the most abundant phyla (approximately 14% in both samples), followed by Actinobacteria (approximately 7%) and Acidobacteria (approximately 5–6%). Collectively, algae and all of these described taxa of bacteria accounted for >90% of the sequences recovered from these biocrusts.

**FIGURE 7:**
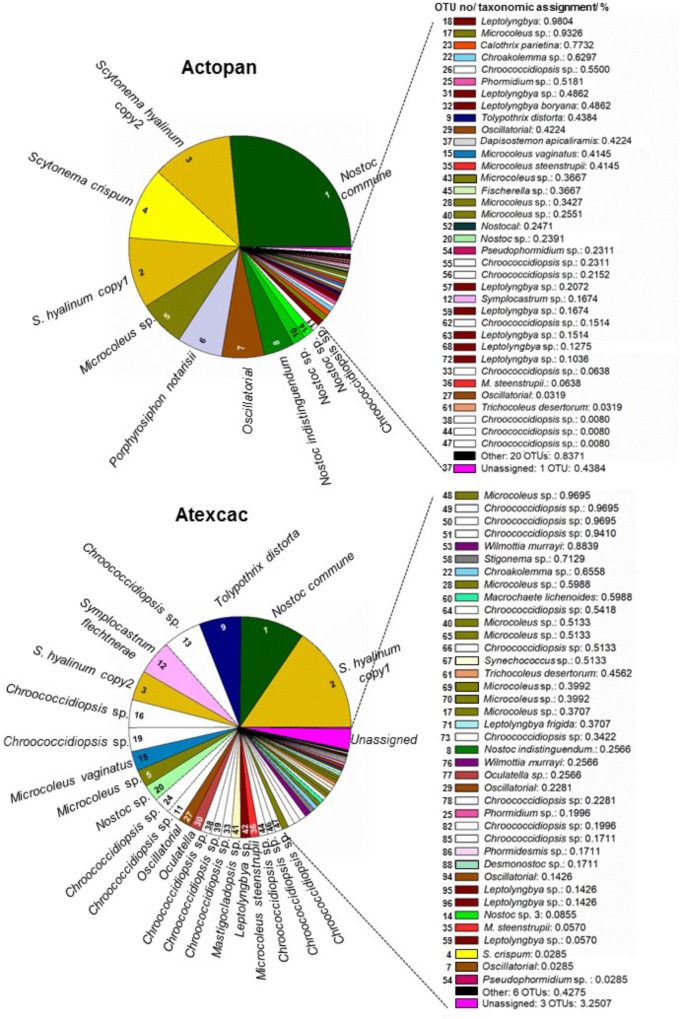
Cyanobacterial community composition from the studied biocrusts. The relative abundance of the OTUs is represented in order of abundance, by a color and the corresponding number (see Table 3 for number, taxonomic assignment and colors).

**FIGURE 8:**
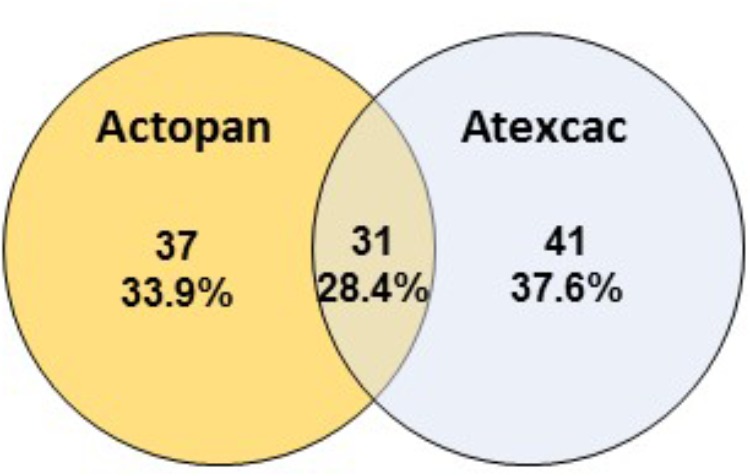
Venn diagram showing overlap of the OTUs among the studied biocrusts.

Regarding cyanobacteria, clear differences were found in their taxonomic composition (Figure 7). The cumulative pie chart shows the distribution of the phylotype counts in which the heterocystous cyanobacteria are by far the most common members at Actopan (69.36%), while at Atexcac, to reach this percentage, it was necessary to combine the heterocystous cyanobacteria (41.03%) with the unicellular/colonial cyanobacteria (31.58%), mostly represented by phylotypes of *Chroococcidiopsis*. Table 3 shows the taxonomic assignments of the OTUs based largely on comparisons with the sequences and the corresponding phenotypes of the isolated cultures in parallel with the microscopic analysis of the biocrust field samples. We could match sequences from the most abundant taxa (corresponding to OTUs 1–16) thanks to the collection of the isolated strains and/or after Blasting and comparing the results with the recorded morphotypes found at the sampling sites (Table 2). Clear differences in the observed taxa at each sampling site were found (Figure 7). Figure 8 summarizes the data for all the OTUs together in a Venn diagram. Only 28.4% of the OTUs were found in both soils, while 33.9% were unique to the Actopan biocrusts and 37.6% were unique to the Atexcac biocrusts. In addition, differences in the relative abundance of the shared cyanobacteria in the samples were also found. *N. commune*, *S. hyalinum*, and *Microcoleus* sp. were more abundant at Actopan than at Atexcac, and some phylotypes, such as those corresponding to *S. crispum*, *N. indistinguendum*, *P. notarisii*, and other Oscillatorial, as well as two OTUs corresponding to *Nostoc* spp. were no present or almost undetectable (less than 1% of abundance) at Atexcac. In contrast, *T. distorta*, *S. flechtnerae*, *M. vaginatus Nostoc* sp., *Oculatella* sp., and several OTUs corresponding to the *Chroococcidiopsis* presented higher abundances at Atexcac than at Actopan, with the percentages of the abundance in the second locality lower than 1%. Interestingly, some of the heterocystous cyanobacteria, although were present in low abundance, were present at only one of the sampling sites: *C. parietina*, *D. apicaliramis*, and other *Nostocal* were present only in Actopan, while *Mastigocladopsis* sp., *Stigonema* sp., *Macrochaete* sp., and *Desmonostoc* sp. were present only at Atexcac (Figure 7).

**Table 3:** Taxonomic assignments of OTUs.

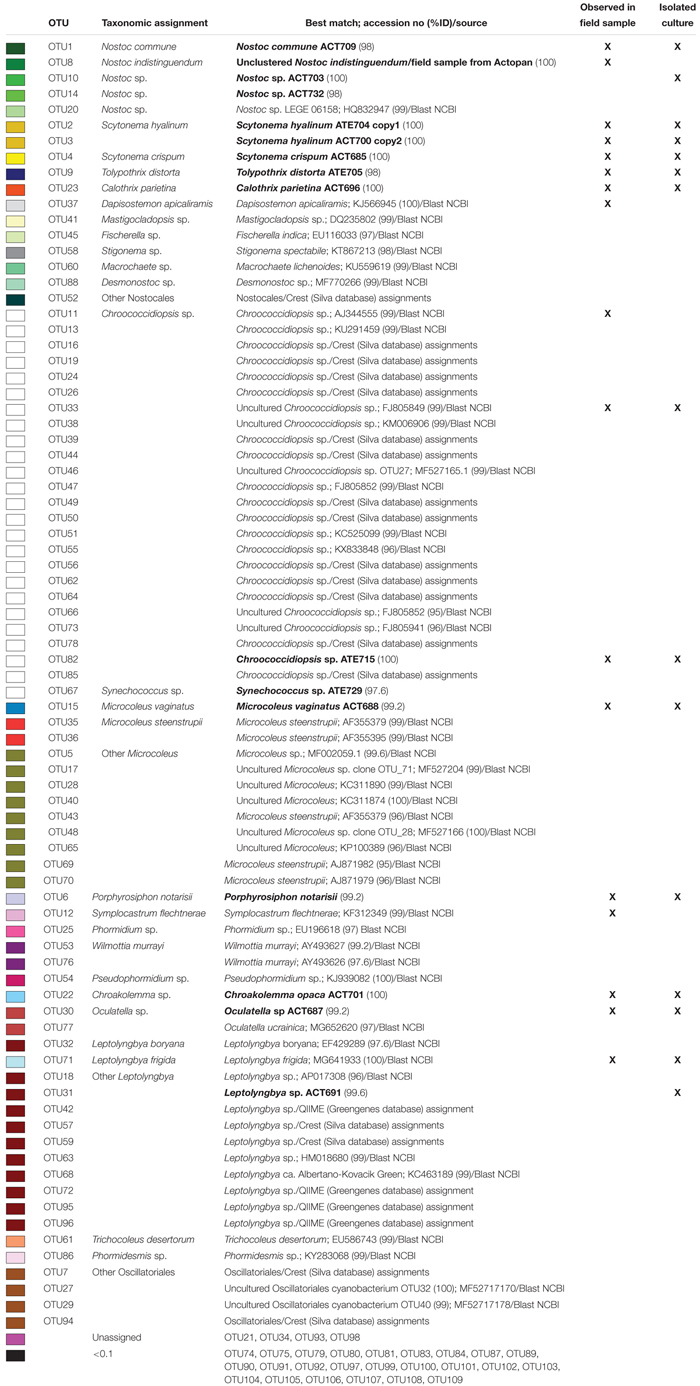

*Isolated strains from this study are in bold. Colors correspond to those in Figure 7.*

### Sensitivity of Biocrust Isolated Cultures to Extreme Heat and Desiccation

Since climatic differences were found at the studied sampling sites, a series of bioassays were carried out to test the sensitivity of the cultured strains to extreme conditions. Table 4 shows (i) the survival of 23 isolated cultures to extreme heat over 1 month, (ii) the subsequent response of the cultures to extreme heat and a year-long desiccation treatment, and (iii) the resilience of the cultures, following these extreme conditions, after the addition of culture medium to the desiccated cells. As expected, the majority of the cultures did not resist extreme heat, showing clear chlorosis within a range of 10–12 days for unicellular/colonial cyanobacteria and 10–20 days for filamentous non-heterocystous forms, except *Kamptonema* sp. which showed clear chlorosis starting on the fourth day of treatment. *Leptolyngbya* sp. survived after 25 days at 40°C but did not resist additional desiccation. Interestingly, the isolated strain belonging to the new genus *Chroakolemma* was the only non-heterocystous strain that survived the entire time tested. However, a variety of responses to extreme heat and desiccation were found for the heterocystous cyanobacteria. *S. crispum* and two strains of *S. hyalinum* and *C. parietina* survived the entire test period, but *T. distorta* did not survive after 19 days of culture at 40°C. Both *Nostoc* strains (*N. commune* and *Nostoc* sp.) survived 25 days of extreme heat but showed clear chlorosis after desiccation and extreme heat, and surprisingly recovered the pigmentation after addition of culture medium at room temperature showing resilience following heat and desiccation disturbance (Table 4).

**Table 4:** Survival of cyanobacterial cultures over 25 days at 40°C, subsequent response of cultures to a year-long desiccation treatment at 40°C, and later survival following the addition of culture medium to the desiccated cells and maintained to room temperature.

	Survival at 40°C (days)												Survival to desiccation at 40°C	Survival after rehydration
			
Isolated strain	3	4	5	6	10	12	14	17	19	20	24	25		
*Scytonema hyalinum* ACT695	+	+	+	+	+	+	+	+	+	+	+	+	+	+
*Scytonema hyalinum* ATE698	+	+	+	+	+	+	+	+	+	+	+	+	+	-
*Scytonema hyalinum* ACT699	+	+	+	+	+	+	+	+	+	+	+	+	+	+
*Scytonema hyalinum* ATE704	+	+	+	+	+	+	-	-	-	-	-	-	-	-
*Scytonema hyalinum* ACT711	+	+	+	+	-	-	-	-	-	-	-	-	-	-
*Scytonema crispum* ACT685	+	+	+	+	+	+	+	+	+	+	+	+	+	+
*Nostoc* sp. ACT703	+	+	+	+	+	+	+	+	+	+	+	+	-	+
*Nostoc commune* ACT709	+	+	+	+	+	+	+	+	+	+	+	+	-	+
*Tolypothrix distorta* ATE705	+	+	+	+	+	+	+	+	-	-	-	-	-	-
*Calothrix parietina* ACT696	+	+	+	+	+	+	+	+	+	+	+	+	+	+
*Microcoleus vaginatus* ACT688	+	+	+	+	+	+	-	-	-	-	-	-	-	-
*Kamptonema* sp. ACT692	+	-	-	-	-	-	-	-	-	-	-	-	-	-
*Porphyrosiphon notarisii* ACT693	+	+	+	+	-	-	-	-	-	-	-	-	-	-
*Chroakolemma opaca* ACT686	+	+	+	+	+	+	+	+	+	+	+	+	+	+
*Oculatella* sp. ACT687	+	+	+	+	+	+	+	+	+	+	+	+	-	-
*Oculatella atacamensis* ATE710	+	+	+	+	+	+	+	+	+	-	-	-	-	-
*Leptolyngbya frigida* ACT684	+	+	+	+	+	+	+	-	-	-	-	-	-	-
*Leptolyngbya* sp. ACT689	+	+	+	+	+	+	+	+	+	+	+	-	-	-
*Leptolyngbya* sp. ACT691	+	+	+	+	+	+	+	+	+	+	+	+	-	-
*Schizothrix* sp. ACT690	+	+	+	+	+	+	+	+	+	-	-	-	-	-
*Chroococcidiopsis* sp. ATE707	+	+	+	+	+	-	-	-	-	-	-	-	-	-
*Chroococcidiopsis* sp. ATE715	+	+	+	+	+	-	-	-	-	-	-	-	-	-
*Synechococcus* sp. ATE729	+	+	+	+	-	-	-	-	-	-	-	-	-	-


## Discussion

The distribution of cyanobacterial species and their abundance is of crucial importance for understanding recent and ancient environmental dynamics. However, data about cyanobacterial species distribution are fragmented and strongly biased depending on the work carried out on a certain continent (Büdel et al., 2016). Factors influencing cyanobacterial distribution have been discussed for a long time, and climate and geography have been the main focus of attention. However, recent studies addressed the importance of soil texture on the growth of different cyanobacteria on the soil, in inoculation experiments (Rozenstein et al., 2014; Chamizo et al., 2018); whereby in areas of high fine soil grains content, a high biocrust cover was found (Belnap et al., 2014). The fact that our study area is dominated by fine-textured soils can explain why we found an extensive biocrust cover, similar to those found in previous studies (Williams et al., 2013; Belnap et al., 2014), and a high cyanobacterial diversity.

Molecular investigations through next-generation sequencing culture-independent approaches or by sequencing inserts of clone libraries have shown differences in cyanobacterial diversity depending on the geographical region. Community composition of microbial phototrophs in arid soil biocrusts of the southwestern United States showed that this region was dominated by the bundle-forming, non-heterocystous cyanobacteria *Microcoleus vaginatus* and *Microcoleus steenstrupii*; the former mostly dominated in the northern locations, and the second mostly dominated in the southern latitudes (Garcia-Pichel et al., 2013). In Artic biocrusts, cyanobacterial communities were dominated by sequences related to the form-genera *Leptolyngbya, Calothrix, Coleofasciculus, Oscillatoria, Stigonema, Microcoleus*, and *Phormidium* (Pushkareva et al., 2015). In southern African biocrusts, in addition to typical genera found in biocrusts, such as *Microcoleus, Phormidium*, *Tolypothrix*, and *Scytonema*, sequences corresponding to the genera *Leptolyngbya, Pseudanabaena, Oscillatoria*, and *Schizothrix* were also found (Dojani et al., 2014). Analysis of the cyanobacterial diversity of western European biocrusts along a latitudinal gradient demonstrates that all the sites, except Spain, included taxa unique to the surveyed localities, but all the sites were dominated by *Leptolyngbya*, *Phormidium*, and a cyanobacterium with no matches in the databases (Williams et al., 2016). Our previous results of biocrusts from a latitudinal and climatic gradient in Spain showed a general dominance of *Microcoleus vaginatus* and *Microcoleus steenstrupii*, although differences between the locations were also found, wherein some heterocystous cyanobacteria, such as *S. hyalinum*, appeared to be dominant in developed biocrusts from the warmest and driest southwest locations (Muñoz-Martín et al., 2019).

The results from the present study revealed clear differences regarding the aforementioned studies. In addition, it is remarkable that although these localities are relatively close and belong to the same desert system (Chihuahuense), the cyanobacterial species composition and abundance are very different between the localities. The taxonomic assignment of the sequences revealed a clear dominance of the heterocystous cyanobacteria in Actopan (Hidalgo state), while in Atexcac (Puebla state), although the Nostocales were also abundant, unicellular/colonial cyanobacteria, mostly *Chroococcidiopsis* spp., represented almost a third of the cyanobacteria present. The Atexcac location harbored greater cyanobacterial diversity than Actopan; in Actopan, the 83% of the cyanobacterial phylotypes corresponded to 8 OTUs, while in Atexcac, this percentage was distributed among 42 OTUs. To the best of our knowledge, our study is the first to show such great cyanobacterial diversity in biocrusts. This high level of diversity suggests a greater ecosystem complexity than previously appreciated. Differences in the cyanobacterial populations in the studied biocrusts compared to other crusts in Mexico were also found. The microscopic analysis of biocrusts from the close Tehuacán Valley, also in the Puebla state, showed that the most common species that were present at the studied sites were *Scytonema javanicum, Microcoleus paludosus*, and *Chroococcidiopsis* sp., while *Nostoc* sp., *Schizothrix* sp., and *Aphanocapsa* sp. were found with a lower frequency, and *Gloeocapsa* sp. was the only rare species of cyanobacteria (Rivera-Aguilar et al., 2006).

Within heterocystous forms, the genera *Nostoc* and *Scytonema* were dominant at both studied locations, which may have relevant ecological implications for the nitrogen cycle and other ecosystem services. *Nostoc* spp. are considered important components of the nitrogen-fixing community in nutrient-poor soils worldwide (Dodds et al., 1995). In addition, macroscopic *Nostoc* colonies, found usually on the surface (Belnap and Lange, 2003) as well as the *Scytonema* filaments, undergo intense environmental stresses. These include high daytime temperatures during the summer, low temperatures during the night in the winter, high visible and UV radiation, and frequent hydration-dehydration cycles, which are characteristic of the conditions found at our sampling locations. The aforementioned cyanobacteria have therefore had to develop adaptive structural and physiological mechanisms to live in these places (Hu et al., 2012). One of these mechanisms for the cyanobacteria inhabiting exposed near-surface soils, is to produce photoprotective accessory pigments, such as scytonemins, mycosporine-like amino acids and carotenoids, which increase in content with solar radiation (Bowker et al., 2002), and reduce the amount of UVR damage accrued when cells are desiccated and metabolically inactive or dormant, when temperatures are suboptimal or freezing (Castenholz and Garcia-Pichel, 2012). Thus, scytonemin-producing cyanobacteria found, play an important role in protecting soil from solar radiation, shading the surrounded community and allowing colonization by other species (Garcia-Pichel and Castenholz, 1991; Singh et al., 2010).

*Chroococcidiopsis* spp., found in high abundance in Atexcac location, although typically considered hypolithic organisms, are also often found at the soil surface, surviving prolonged desiccation and developing sheaths containing pigments for UV protection (Dillon et al., 2002). In these extreme conditions, usual in this location, adaptation is also related to the secretion of a copious hygroscopic extracellular polymeric substance that enables cyanobacteria to cope with prolonged moisture deficit in hot deserts (Pointing, 2016).

The capacity to adapt to different environmental conditions can be distinct and depends on the attributes of cyanobacteria. Differences in climatic conditions between the studied sites were found and to investigate the possible interrelation between these climate conditions and differences in the cyanobacterial community composition, we analyzed the specific sensitivity of the isolated biocrust cultures to heat and desiccation. The results clearly showed differences between the analyzed strains which might be responsible for generating the differences in composition found. For instance, *S. hyalinum* and *N. commune*, which were able to survive to extreme conditions in the bioassays, presented higher abundances at the Actopan location, where a maximum temperature of 47°C was reached, than at Atexcac, where the maximum temperature was just 36°C. Much of the success of *Nostoc* in dryland habitats is related to its ability to remain desiccated for months, years, or even several decades (Hu et al., 2012) and to fully recover its metabolic activity within hours to days after rehydration with liquid water (Dodds et al., 1995). In fact, it has been found that *N. commune* from terrestrial habitats in China recovered after a drought period of 2 years, with reactivation of respiration, photosynthesis, and nitrogen fixation (Scherer et al., 1984). Evidence has shown that the development of specialized cells, such as akinetes (spore-like) which are cell survival stages that are known only in heterocystous cyanobacteria, leads to higher tolerance to dryness than in other stages (Hu et al., 2012). Therefore, the development of akinetes in *Nostoc* cultures can explain the observed survival after a year of desiccation at 40°C. Additionally, desiccation provides some protection to high temperature for biocrust cyanobacteria (Lan et al., 2014) which could also explain the resilience to this disturbance.

Understanding differences in the cyanobacterial diversity living in dryland areas remains essential since these biota play a critical role in ecosystem functioning. In addition to the geographic patterns of distribution discussed above, the heterogeneity of microenvironments or microclimatic gradients can also influence cyanobacterial diversity. Therefore, in our study, the nature of the cyanobacterial community structure seems to depend on these specific characteristics rather than the geographic location of the study sites. For a long time, some cyanobacteria have been reported as unique and endemic to specific sites, such as hot or polar environments (e.g., Castenholz, 1996; Taton et al., 2003). The question of endemism of cyanobacteria remains unsettled, and currently accepted theories about microbial biogeography assume that most microorganisms are cosmopolitan and ubiquitous (Fenchel and Finlay, 2004; Foissner, 2008). The paradigm “everything is everywhere, the environment selects” (Beijerinck, 1913; Becking, 1934) has been a starting point for studies of prokaryotic biodiversity and their biogeographical patterns (De Wit and Bouvier, 2006 and references therein). However, recent studies dispute the idea that ‘everything is everywhere,’ and the claim that ‘the environment selects’ implies that different contemporary environments maintain distinctive microbial assemblages (Martiny et al., 2006). Komárek (2015) found specific endemic Antarctic cyanobacterial units, inconsistent with the cosmopolitan hypothesis. Furthermore, several authors encourage the theory of cyanobacterial endemism, reporting unique distributions of some taxa. Sherwood (2004, 2007) and Sherwood et al. (2015) characterized Hawaiian freshwater and terrestrial cyanobacteria, revealing high diversity and numerous cyanobacterial species that are believed to be endemic to the Hawaiian Islands. Kastovsky et al. (2016) described two new cyanobacterial species, presumably endemic taxa of the Chimantá Massif, Venezuela. Analysis of the cyanobacteria from hot water springs of the North-Western Himalayas harbored endemic cyanobacterial species (Singh et al., 2018). Lastly, in comparing the cyanobacteria within the biocrusts of the Arctic, Antarctic, and European Alpine sites, the discovery of a new *Oculatella* species, through the polyphasic approach, enhanced arguments for cold-assigned cyanobacterial endemism (Jung et al., 2018). The results from the present study support this view, whereby phylogenetic analysis revealed novel phylotypes not previously found with some of them present in high abundances (see, e.g., OTU 7, and those taxa corresponding mainly to *Chroococcidiopsis* spp.). Mexico contains a great diversity of topography and climate because of its complex geology, with the existence of a fair number of regions that behave as true ecological islands and peninsulas; these climatic and topographical attributes have led to the recognition of a large proportion of endemic genera, most of them related to the degree of climatic aridity (Rzedowski, 1991; Rodrigues et al., 2004) which is a trait that can be reflected in the cyanobacteria present as well. In fact, although the two sampling sites belong to the Chihuahuan Desert, they are separated by the Trans-Mexican Volcanic Belt, a forested cordillera lacking biocrusts that acts as a high elevation barrier to the dispersal of plant and animal species. Thus, our sites harbored cosmopolitan cyanobacteria, such as the typical taxa generally found in biocrusts (e.g., *N. commune, S. hyalinum, T. distorta*) but also presented novel biocrust-associated phylotypes that could be endemic. Further analysis of the biogeographical relationships and extending the sampling sites comprehensively throughout the world will determine if the current putative endemic cyanobacteria reflect the gaps in our knowledge of cyanobacterial diversity or if there is true endemism.

## Author Contributions

IB-A and PM designed the study. IB-A collected the biocrust samples and performed laboratory work. IB-A and GM performed the microscopic study. IB-A, MM-M, and PM performed the bioinformatics analysis of sequence data. The first draft of this manuscript was written by PM and all co-authors contributed to improve it.

## Conflict of Interest Statement

The authors declare that the research was conducted in the absence of any commercial or financial relationships that could be construed as a potential conflict of interest.

## Footnotes

1 http://smn.cna.gob.mx/es/

2 https://www.inegi.org.mx/

3 http://www.conabio.gob.mx/informacion/gis/

4 http://clicom-mex.cicese.mx

5 http://www.ncbi.nlm.nih.gov/blast
